# Influence of *Plantago ovata* husk (dietary fiber) on the bioavailability and other pharmacokinetic parameters of metformin in diabetic rabbits

**DOI:** 10.1186/s12906-017-1809-x

**Published:** 2017-06-07

**Authors:** Raquel Díez, Juan José García, María José Diez, Matilde Sierra, Ana M. Sahagun, Nélida Fernández

**Affiliations:** Pharmacology, Institute of Biomedicine (IBIOMED), University of León, 24071 León, Spain

**Keywords:** Pharmacokinetic, Metformin, Rabbits, Fiber, *Plantago ovata* Husk

## Abstract

**Background:**

Metformin is an oral hypoglycemic agent frequently used in patients with type 2 diabetes. In this study, we have investigated the influence of the dietary fiber *Plantago ovata* husk on the pharmacokinetics of this drug when included in the diet, as well as when administered at the same time as metformin.

**Methods:**

Six groups of 6 rabbits were used. Groups 1 to 3 were fed with standard chow and groups 4 to 6 with chow supplemented with fiber (3.5 mg/kg/day). Groups 1 and 4 received metformin intravenously (30 mg/kg). Groups 2 and 5 received metfomin orally (30 mg/kg), and number 3 and 6 were treated orally with metformin (30 mg/kg) and fiber (300 mg/kg).

**Results:**

The changes caused by the inclusion of fiber in the feeding were more important in groups that received oral metformin. In this way, metformin oral bioavailability showed an increase of 34.42% when rabbits were fed with supplemented chow.

**Conclusions:**

*Plantago ovata* husk increased the amount of absorbed metformin when included in the diet (significant increase in AUC), and delayed its absorption when administered at the same time (significant increase in t_max_).

## Background

The prevalence of type 2 diabetes has increased over the last decades and continues to rise. Oral antidiabetic agents are the main drugs used to treat type 2 diabetes, and currently, there are six distinct classes of hypoglycemic agents available.

Metformin features as a current first-line pharmacological treatment in almost all guidelines and recommendations worldwide. Pharmacologically belongs to biguanide class of antidiabetic drugs. The main action of metformin was based on the increased transport of glucose across the cell membrane of skeletal muscle. Studies suggested that reducing hepatic gluconeogenesis was the most important mechanism for developing hypoglycemic activity [1, 2]. Gluconeogenesis suppression involves several substrates: lactate, pyruvate, glycerol, and aminoacids. Metformin increases intramitochondrial calcium levels, which modulate mitochondrial respiration. In insulin-sensitive tissues (liver, skeletal muscle and adipocytes) the drug increased the activity of tyrosine kinase receptors of insulin, which stimulates the transporter GLUT4, increasing traffic transmembrane glucose, insulin levels and glucose fasting in diabetic and nondiabetic patients [2].

Besides these important antidiabetic effects, which have been manifest utility in patients with type 2 diabetes [3] and in preventing it in patients with obesity and insulin resistance [4], metformin has actions on body weight [5], serum lipids level [6], blood pressure [7], vascular effect [8], nitroxidative and oxidative stress magnitude [9], and inflammation, arterial structure and vasoprotection [10–12].

AMP activated protein kinase (AMPK) controls the metabolism of glucose and lipids [13, 14] in many tissues of mammals. Metformin is able to activate AMPK in rat hepatocytes, and as a result reduces the activity of acetyl-CoA carboxylase (ACC), which leads to an increased fatty acids β-oxidation into the mitochondria and inhibition of glucose production [15, 16]. The same process occurred in bovine aortic cells [17], in skeletal muscle [18] and mouse ovary [19].

Knowledge the pharmacokinetics of any compound was essential to establish the optimal treatment regimen. Metformin pharmacokinetics has been fully documented in humans [20–28] but less in animal species such as rats [31–33], cats [34, 35] and horses [36].

The increasing intake of purified fibers like psyllium or ispaghula husk (the husk of the seeds of *Plantago ovata*) in the treatment of diseases makes it necessary to know how these fibers interact with drugs used simultaneously. However, these studies are scarce, and their results variable. Most studies suggest that *Plantago ovata* husk interacts with drugs while others did not find any interaction [37]. It has been shown that the bioavailability of drugs when are orally administered depends on the processes of absorption and plasma clearance, and both can be affected by the presence of certain dietary components in the gastrointestinal tract [38, 39]. Psyllium is also a fiber with hypoglycemic properties [40–44], so it is possible it can contribute to reduce glucose levels when administrated with metformin. The aim of the present study was to evaluate the influence of *Plantago ovata* husk in the bioavailability and other pharmacokinetic parameters of metformin in diabetic rabbits. Modifications caused by the presence of fiber in the diet were determined, and also those produced when psyllium and metformin were orally administered at the same time. A correlation with glucose and insulin level was also established.

## Methods

### Study design

All studies were performed in accordance with the Spanish Regulations for the handling and use of laboratory animals (RD 53/2013). Taking into account in the study the “3Rs” minimum number of animals and duration of observation required to obtain consistent data were employed. All experimental protocols were approved in advance by the Animal Care Committee at the University of León.

Thirty-six healthy male New Zealand white rabbits weighting 2.650–3.240 kg were used. They were maintained in a restricted access room in the Animal Care Facility at the University of León (Spain), in individual metal cages which allowed the isolation of faeces in a lower container to avoid coprophagia. The environmental conditions were temperature 19 ± 2 °C, relative humidity 55 ± 10% and a 12 h light-dark cycle. Rabbits were maintained under these conditions at least 1 week before the assay, with free access to water and standard laboratory chow. The rabbits were randomly divided into six groups of 6 rabbits each. All the animals of the first, second and third group received standard chow and the rabbits of the fourth, fifth and sixth group received standard chow supplemented with fiber, providing a daily 3.5 mg/kg dose of *Plantago ovata* husk (Plantaben®, Rottapharm S.L., Barcelona, Spain) during 35 days. Diabetes was induced by the intravenous administration of alloxan (80 mg/kg) dissolved in 10 mL NaCl solution in the marginal ear vein on day 14 of the study.

On day 35, the rabbits in the first and forth group received metformin 30 mg/kg intravenously. Metformin was administered as a solution in a mixture of saline:ethanol (9:1, *v*/v) into the marginal ear vein. This administration was carried out to establish metformin elimination kinetics and bioavailability.

Animals of the second and the fifth group were treated with 30 mg/kg of metformin solution. The drugs were administered by gastric intubation. A total of 50 mL water was used for administration and cannula cleaning.

Finally, groups number three and six received fiber (300 mg/kg) in addition to metformin (30 mg/kg). Metformin solution and fiber dispersed in water were administered by gastric intubation and a total of 50 mL of water was used for administration and cannula cleaning.

The animals were anaesthetized with sodium pentobarbital (30 mg/kg body weight, i.v.) and the left carotid artery cannulated with a silicone catheter. Metformin and fiber administration was carried out after total recovery from anaesthesia was achieved.

### Pharmacokinetic studies

Blood samples (3 mL) were obtained from the carotid artery through the cannula into heparinized containers before and at 5, 10, 20, 30, 60, 90, 120, 180, 240 and 300 min, after drug and fiber administration. Immediately after collection, plasma was separated by centrifugation and stored at −20 °C until analyzed.

Metformin extraction from plasma samples was carried out using solid-phase extraction (SPE) and HPLC with ultraviolet detection for quantification. Neither heparin nor pentobarbital interfered on the assay.

Pharmacokinetic analysis was performed based on a non-compartmental description of the data observed. Maximum plasma metformin concentration (C_max_) and the time to reach maximum concentration (t_max_) were read directly from the individual plasma concentration-time curves. The WinNonlin computer program and a formula described by Gibaldi and Perrier (1982) were used to calculate the model-independent pharmacokinetic parameters. These parameters were elimination rate constant (λ), area under the plasma concentration-time curve (AUC), clearance (Cl/F), volume of area (V_a_/F), volume of distribution at steady state (V_ss_/F), half-life associated with the λ phase (t_1/2λ_), area under the first moment curve (AUCM) and mean residence time (MRT). The bioavailability of metformin (F%) was calculated by dividing the mean AUC by the value of the mean i.v. AUC after the administration of metformin alone.

### Glucose and insulin determinations

To evaluate glucose and insulin levels, a 3 g glucose load was given after drug administration to the groups of rabbits previously described. Blood samples (1 mL) were obtained from the marginal ear vein, using an intravenous catheter, before (time 0) and 30, 60, 120, and 180 min after glucose load was given. Immediately after collection, plasma were separated by centrifugation and stored at −20 °C until analyzed.

Glucose and insulin was determined in the blood samples. Glucose determination was carried out using a biochemical autoanalyzer (Cobas Integra 400) and insulin by a radiometric method using a kit (Mercodia Ultrasensitive Insulin ELISA, Biosource Europe, SA). The means, standard deviations, and coefficients of variation were computed from the results.

### Statistical analysis

All the pharmacokinetic parameters were calculated for each animal, and the data were presented as arithmetic mean ± standard deviation (mean ± SD). The t Test was used to evaluate differences between parameters after logarithmic transformation of the values, except for t_max_. This parameter was compared by using the Mann-Whitney Test.

The glucose and insulin data obtained in the groups of rabbits that received standard chow or supplemented with *Plantago ovata* husk were calculated for each animal, and the data were presented as arithmetic mean ± standard deviation (mean ± SD). Data were analyzed by the Shapiro-Wilk Test (to determine normality) and Levene Test (to determine uniformity invariance). When the data were normal and with uniformity in variance, the t Test was used. When the data were no normal and with or without uniformity invariance, the Mann-Whitney Test was used.

*P* ≤ 0.05 was used as the level of significance for all analyses. All analyses were performed by using SPSS Statistics 21.0 for Windows.

## Results

### Intravenous route

**Fig. 1:**
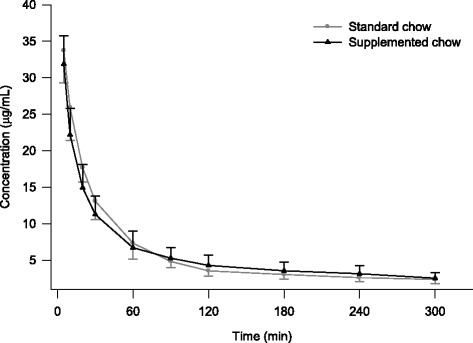
Mean plasma concentrations of metformin in diabetic rabbits after the intravenous administration of 30 mg/kg

Mean plasma concentrations of metformin as a function of time following the administration of 30 mg/kg to rabbits shown in Fig. 1. We observed that the initial concentrations are slightly higher in rabbits fed with standard chow. However, the final concentrations are higher in the group of animals fed with supplemented chow.

The pharmacokinetic parameters derived from non-compartmental analysis are summarized in Table 1.

**Table 1:** Non-compartmental pharmacokinetic parameters obtained for metformin after its intravenous administration (30 mg/kg) to diabetic rabbits (*n* = 6)

Parameters	Standard chow		Supplemented chow		*p* values
	$\overline{x}$ ± SD	CV (%)	$\overline{x}$ ± SD	CV (%)	
λ (min^−1^)	0.0022 ± 0.0011	50.04	0.0028 ± 0.0008	28.78	0.233^a^
AUC (μg·min/mL)	3171.9 ± 680.4	21.45	2806.1 ± 471.9	16.82	0.351^a^
Cl (mL/min/kg)	9.86 ± 2.29	23.20	10.99 ± 2.14	19.47	0.351^a^
V_a_ (mL/kg)	5137.7 ± 1839.9	35.81	4301.6 ± 1786.6	41.53	0.382^a^
V_ss_ (mL/kg)	3829.3 ± 1483.7	38.74	3279.1 ± 1174.1	35.81	0.487^a^
t_1/2λ_ (min)	385.9 ± 47.71	47.71	268.5 ± 85.4	31.80	0.220^a^
AUMC (μg·min^2^/mL)	1,411,571.6 ± 915,944.8	64.89	847,182.9 ± 324,044.5	38.25	0.279^a^
MRT (min)	81.70 ± 3.98	4.87	89.81 ± 13.02	14.50	0.198^a^

^a^t Test

We observed that all values are higher in rabbits fed with standard chow, except λ, Cl and MRT. In this case, these parameters (λ, Cl and MRT) were higher in animals fed with supplemented chow with increments of 21.42, 10.28 and 9.03%, respectively. However, there were not significant differences between metformin pharmacokinetic parameters obtained after its intravenous administration in animals fed with standard chow and in those that received the chow supplemented with *Plantago ovata* husk: λ, AUC, Cl, V_a,_ V_ss,_ t_1/2λ,_ AUMC and MRT.

Glucose and insulin concentrations obtained at the different sampling times are shown in Table 2.

**Table 2:** Glucose and insulin concentrations obtained after the administration of an oral 3 g glucose load to diabetic rabbits (*n* = 6) treated with an intravenous metformin dose of 30 mg/kg

Time (min)	Glucose (mg/dL)			Insulin (mUI/L)		
	Standard chow (group 1)	Supplemented chow (group 4)	*p* values ^a^	Standard chow (group 1)	Supplemented chow (group 4)	*p* values^a^
0	399.9 ± 81.72	375.7 ± 152.0	0.737	0.616 ± 0.561	0.801 ± 0.464	0.547
30	461.6 ± 28.41	446.9 ± 154.2	0.824	0.990 ± 0.779	1.227 ± 0.814	0.617
60	496.4 ± 46.99	497.0 ± 122.1	0.991	1.297 ± 0.853	1.718 ± 1.202	0.500
120	552.3 ± 39.15	497.4 ± 102.5	0.248	1.000 ± 0.274	1.892 ± 1.186	0.103
180	471.7 ± 81.52	457.6 ± 117.4	0.814	0.573 ± 0.238	1.432 ± 0.940	0.055

^a^t Test

Glucose was slightly higher in the group fed with standard chow (group 1) than in those animals that received chow supplemented (group 4) with *Plantago ovata* husk. Insulin concentrations were higher in those animals that received fiber supplemented chow (group 4) at all the sampling times than in the group fed with standard chow (group 1). However, no significant differences were observed in glucose and insulin concentrations.

### Oral route

**Fig. 2:**
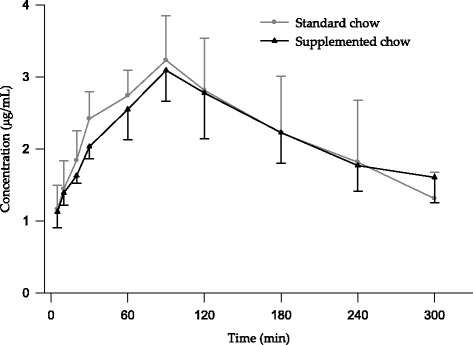
Mean plasma concentrations of metformin in diabetic rabbits after the oral administration of 30 mg/kg

The plot of mean plasma metformin concentrations as a function of time after oral administration (30 mg/kg) for the two groups studied are shown in Fig. 2. We observed that the concentrations are slightly higher in rabbits fed with standard chow, except in the range 240–300 min.

The non-compartmental pharmacokinetic parameters obtained after the administration of metformin with standard or supplemented chow are summarized in Table 3.

**Table 3:** Non-compartmental pharmacokinetic parameters obtained for metformin after its oral administration (30 mg/kg) to diabetic rabbits (*n* = 6)

Parameters	Standard chow		Supplemented chow		*p* values
	$\overline{x}$ ± SD	CV (%)	$\overline{x}$ ± SD	CV (%)	
λ (min^−1^)	0.0046 ± 0.0007	14.43	0.0029 ± 0.0011	39.32	0.014^a^
AUC (μg·min/mL)	960.6 ± 230.9	24.03	1296.2 ± 331.5	25.57	0.048^a^
C_max_ (μg/mL)	3.23 ± 0.614	19.01	3.09 ± 0.425	13.77	0.694^a^
t_max_ (min)	90.0 ± 0.00	-	90.0 ± 0.00	-	1.000^b^
Cl/F (mL/min/kg)	32.70 ± 7.41	22.67	24.39 ± 5.99	24.55	0.062^a^
V_a_/F (mL/kg)	7374.6 ± 2253.7	30.56	9074.9 ± 2680.1	29.53	0.252^a^
V_ss_/F (mL/kg)	4416.9 ± 758.8	17.18	3445.3 ± 702.4	20.39	0.046^a^
t_1/2λ_ (min)	155.3 ± 27.13	17.48	271.6 ± 100.9	37.13	0.014^a^
AUMC (μg·min^2^/mL)	245,796.4 ± 65,691.2	26.73	571,150.5 ± 320,022.7	56.03	0.015^a^
MRT (min)	136.6 ± 8.51	6.23	142.5 ± 6.52	14.50	0.199^a^
MAT (min)	54.95		52.72		
F (%)	30.29		46.19		

^a^t Test; ^b^Mann-Whitney Test

The pharmacokinetic parameters λ, AUC, V_ss_/F, t_1/2λ_ and AUMC obtained for metformin showed significant differences between animals fed with standard or supplemented chow. λ and V_ss_/F increased 36.99% and 22.0%, respectively, when animals were fed with standard chow. On the other hand, AUC, t_1/2λ_ and AUMC increased 34.94%, 42.82% and 59.97%, respectively, when rabbits were fed with supplemented chow. The other pharmacokinetic parameters evaluated (C_max_, t_max_, Cl/F, V_a_/F and MRT) showed no significant differences. The mean bioavailability of metformin was approximately 30.29% for rabbits fed with standard chow and 46.19% for animals fed with supplemented chow, this represents an increase of 34.42%.

In Table 4 we can observe the glucose and insulin concentrations obtained at the different sampling times. The maximum concentration of glucose is reached at 60 min. From this time on, the glucose values were lower in group 5 (supplemented chow) although there were no significant differences. The changes caused by the inclusion of fiber in the feeding were more important in these groups (2 and 5) than in the other ones evaluated (groups 1, 3, 4 and 6). The higher levels of insulin we found in group 5 and also detected the highest insulin concentrations of all groups studied, with significant differences at 120 and 180 min.

**Table 4:** Glucose and insulin concentrations obtained after the administration of an oral 3 g glucose load to diabetic rabbits (*n* = 6) treated with an oral metformin dose of 30 mg/kg

Time (min)	Glucose (mg/dL)			Insulin (mUI/L)		
	Standard chow (group 2)	Supplemented chow (group 5)	*p* values^a^	Standard chow (group 2)	Supplemented chow (group 5)	*p* values^a^
0	326.5 ± 85.76	329.2 ± 64.50	0.953	0.492 ± 0.412	1.242 ± 0.789	0.066
30	453.9 ± 90.13	458.7 ± 108.2	0.935	1.681 ± 1.288	2.930 ± 1.686	0.180
60	515.0 ± 87.21	474.2 ± 96.59	0.272	1.823 ± 1.385	3.883 ± 2.227	0.083
120	501.9 ± 63.41	426.4 ± 112.5	0.233	1.755 ± 1.316	4.964 ± 2.601	0.022
180	453.9 ± 88.45	395.8 ± 115.5	0.511	1.249 ± 1.142	3.617 ± 1.859	0.024

^a^t Test

### Oral route with *Plantago ovata* husk

**Fig. 3:**
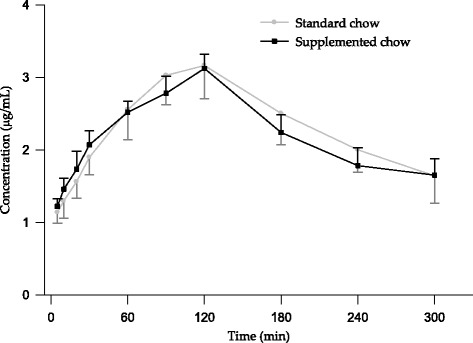
Mean plasma concentrations of metformin in diabetic rabbits after its oral administration (30 mg/kg) with *Plantago ovata* husk (300 mg/kg)

Figure 3 includes the mean values of plasma metformin concentrations vs. time obtained in the two groups studied. This figure shows that, the first 50 min, metformin concentrations were lower for standard chow group although from this point time on drug concentrations were always higher in animals belonging to this group.

Table 5 includes the non-compartmental pharmacokinetic parameters obtained after the administration of metformin with fiber for each group of rabbits. The higher increases were found in t_1/2λ_ and AUMC (43.61 and 54.02%, respectively) for animals that received supplemented chow, although there were no significant differences. No significant differences were found in other parameters λ, AUC, C_max_, t_max_, Cl/F, V_ss_/F and MRT. When comparing the value of AUC obtained after metformin oral administration together with *Plantago ovata* husk with that found after intravenous administration, it can be seen that the bioavailability is 36.73 and 41.52%, after receiving standard or supplemented chow, respectively. This represented an increase of 11.54%.

**Table 5:** Non-compartmental pharmacokinetic parameters obtained for metformin (30 mg/kg) after its oral administration with *Plantago ovata* husk (300 mg/kg) to diabetic rabbits (*n* = 6)

Parameters	Standard chow		Supplemented chow		*p* values^a^
	$\overline{x}$ ± SD	CV (%)	$\overline{x}$ ± SD	CV (%)	
λ (min^−1^)	0.0039 ± 0.0010	25.17	0.0025 ± 0.0014	53.87	0.053^a^
AUC (μg·min/mL)	1164.9 ± 262.1	22.50	1496.9 ± 438.9	29.32	0.160^a^
C_max_ (μg/mL)	3.39 ± 0.290	8.53	3.12 ± 0.197	6.32	0.087^a^
t_max_ (min)^c^	110.0 ± 15.49	14.08	120.0 ± 0.00	-	0.138^b^
Cl/F (mL/min/kg)	26.83 ± 5.81	21.67	21.65 ± 6.88	31.76	0.160^a^
V_a_/F (mL/kg)	7070.8 ± 884.9	12.52	9317.5 ± 1803.8	19.36	0.022^a^
V_ss_/F (mL/kg)	3891.2 ± 731.8	18.81	3080.4 ± 908.7	29.50	0.101^a^
t_1/2λ_ (min)	189.6 ± 44.7	17.48	336.2 ± 152.6	45.40	0.052^a^
AUMC (μg·min^2^/mL)	380,326.9 ± 158,923.7	41.79	827,234.6 ± 560,290.1	67.73	0.093^a^
MRT (min)	145.9 ± 6.37	4.36	143.1 ± 4.07	2.84	0.409^a^
MAT (min)	64.21		56.07		
F (%)	36.73		41.52		

^a^t Test

^b^Mann-Whitney Test

^c^significant differences with the value obtained when metformin was administered alone (Mann-Whitney Test)

If we consider the type of chow received by animals treated with metformin with and without fiber, significant differences were observed in λ, AUC, V_ss_/F, t_1/2λ_ and AUMC. Conversely, there were no significant differences for the other parameters.

When the effect of co-administration of metformin with fiber versus metformin alone was evaluated, we only found significant differences for t_max_ (Mann-Whitney Test, *p* = 0.002).

Finally, as show Table 6, after metformin (30 mg/kg) with *Plantago ovata* husk (300 mg/kg) oral administration the glucose concentrations were higher in the group 3 (fed with standard chow) than in the group 6 (supplemented chow) with significant differences at time 30 and 180 min. Insulin concentrations were much lower in those animals that also received *Plantago ovata* husk in their feeding (group 6) than in the group fed with standard chow (group 3). In this case, we observed significant differences at times 0, 30, 60 and 120 min.

**Table 6:** Glucose and insulin concentrations obtained after the administration of an oral 3 g glucose load to diabetic rabbits (*n* = 6) treated with oral metformin (30 mg/kg) and *Plantago ovata* husk (300 mg/kg)

Time (min)	Glucose (mg/dL)			Insulin (mUI/L)		
	Standard chow (group 3)	Supplemented chow (group 6)	*p* values^a^	Standard chow (group 3)	Supplemented chow (group 6)	*p* values^a^
0	457.7 ± 37.74	400.9 ± 79.04	0.133	0.936 ± 0.238	0.333 ± 0.070	0.000
30	528.7 ± 26.83	471.4 ± 52.61	0.039	1.773 ± 0.876	0.646 ± 0.169	0.011
60	573.0 ± 18.86	508.6 ± 81.48	0.089	3.579 ± 2.399	1.124 ± 0.652	0.036
120	543.0 ± 58.18	489.7 ± 66.09	0.102	1.517 ± 0.538	0.832 ± 0.397	0.031
180	530.2 ± 53.90	407.1 ± 58.62	0.004	1.075 ± 0.534	0.692 ± 0.333	0.167

^a^t Test

## Discussion

Several authors indicate that the pharmacokinetics of metformin after oral administration is well described by a three-compartment open model [22, 29, 34] although most researchers have evaluated its pharmacokinetics using a non-compartment model [25, 27, 31–33, 36, 45, 46]. In this study, we tried a compartmental analysis of the results obtained, but the plasma concentration-time data were not adequately fitted to the classic open compartmental models tested, so finally, we applied a non-compartmental model.

Metformin pharmacokinetics has an absolute oral bioavailability of 40 to 60%, and gastrointestinal absorption is apparently complete within 6 h of ingestion [26]. Clinical trials with metformin have demonstrated decreased bioavailability at higher doses, suggesting saturable intestinal absorption [22, 25, 26, 30]. The relative contributions of transcellular and paracellular transport to the overall transport of metformin (0.05 mM) were estimated to be 9 and 91%, respectively [30].

There are numerous publications of metformin pharmacokinetics in different species but not in the rabbit so our results are going to be compared with those obtained in other models like rats, cats, horses or humans. The mean value for C_max_ determined in the group that received oral metformin was 3.23 μg/mL (standard chow), similar to the obtained by Choi et al. [31], 3.4 μg/mL, after the administration of a higher oral dose of metformin (50 mg/kg) in rats. Mean values reported for this parameter were lower in cats [34]: 1.9 μg/mL after a 25 mg/kg oral dose, and in horses [36]: 0.3 μg/mL after a 6 g oral dose. In humans, data were variable: 3.10 μg/mL with a dose of 1.5 g [22], 1.64 μg/mL with a dose of 550 mg [27], 1.67 μg/mL with a dose of 850 mg [47] and 1.77 μg/mL with a dose of 1 g [46].

Regarding t_max_, this parameter in our study was higher (90 min) than that reported in rats (30 min) [31], in cats (60 min) [34] and in horses (78 min) [36]. In humans, again, data were variable, ranging from 75 min [45], 90 min [22], 180 min [27] to 235 min [47].

The bioavailability of metformin in rats was 34.1% [31], in cats 48% [34] in horses 3.9% [36] and in humans 50–60% [22]. In our study, this parameter was similar to the reported in rats, 30.29%.

On the other hand, we have not found any study about the interaction between metformin and *Plantago ovata* husk. We only found the research of [48], who studied the effect of guar gum on the digestive absorption of metformin in 6 healthy subjects after the administration of 1700 mg metformin with a standard meal. Metformin blood levels showed that when metformin was given together with guar gum there was a reduction in the absorption rate over the first 6 h.

Drug interactions have been the subject of numerous studies, but few of them have been carried out with dietary fiber, being the results obtained variable. *Plantago ovata* husk has the potential for producing both benefits and risks associated with desired and undesired effects when coadministered with drugs [37]. Several studies have demonstrated that this fiber could reduce the absorption of drugs administered at the same time, resulting in subclinical concentrations and abolish the effect [49–52].

Some changes in the absorption pharmacokinetic parameters of metformin after its administration with *Plantago ovata* husk at the same time could be due to a delay in gastric emptying. We think that these changes occur because the fiber forms a highly viscous solution, trapping metformin inside it, with consequent decrease in metformin absorption in the intestine and therefore, lower values of C_max_ are obtained.

Another aspect that could have contributed to the results obtained with the fiber could be an increase in the paracelullar absorption of metformin across the gut wall.

According to the authors [53], the groups of animals that received chow supplemented with *Plantago ovata* husk showed lower concentrations of glucose than those fed with standard chow. The largest decreases in glucose concentrations were found in rabbits fed with supplemented chow and that received oral metformin and *Plantago ovata* husk. This result may indicate that *Plantago ovata* husk offers interesting perspectives to be used as adjuvant in patients with type 2 diabetes treated with metformin. Several studies have reported that the addition of fiber as a supplement causes an improvement in glycemic control [40–44].

The lowering of insulin with increasing dietary fiber intake that was observed in our study is consistent with previous findings [54–56].

As found by Diez-Laiz et al. [53], this study showed a great difference between each rabbit in insulin concentrations. These important interindividual variations were more pronounced in rabbits fed with supplemented chow because baseline values were more higher. This fact can be attributed to the diabetes animal model. A type 2 diabetes was induced, so insulin is still produced but some rabbits are mild diabetic while others showed severe diabetes [57].

## Conclusions

The results of our study indicate that the fiber increases the amount of absorbed metformin and slows the rate of absorption. This effect is reflected significantly in the amount absorbed if the fiber is included in the feed and is administered continuously. There is a while significant delay in t_max_ when the fiber is administered, being the great retard after a single dose of metformin.

Although further studies are necessary, and due to the significant reduction observed in glucose concentrations when rabbits were fed with supplemented chow and fiber was administered at the same time as metformin we think that *Plantago ovata* husk can contribute to decrease glucose levels, in patients with type 2 diabetes in treatment with metformin and therefore, maybe a useful dietary adjunct for the treatment of hyperglycemia. In this way, significant decreases in glucose concentrations were found in several sampling times when *Plantago ovata* husk was administered with metformin.
